# Supplementary material for: mTORC1 signaling facilitates differential stem cell differentiation to shape the developing murine lung and is associated with mitochondrial capacity
Source: Nat Commun. 2022 Nov 25;13:7252. doi: 10.1038/s41467-022-34763-y (PMC9700781; doi:10.1038/s41467-022-34763-y)
Supplement: Supplementary file 1 — Supplementary Information [file 41467_2022_34763_MOESM1_ESM.pdf]

mTORC1 signaling facilitates differential stem cell differentiation to shape the developing murine lung and is associated with mitochondrial capacity

Kuan Zhang[1], Erica Yao[1], Ethan Chuang[1], Biao Chen[1], Evelyn Y. Chuang[1] and Pao-Tien Chuang[1]

[1]Cardiovascular Research Institute, University of California, San Francisco, CA 94158, USA

Correspondence and requests for materials should be addressed to Pao-Tien Chuang (email: pao-tien.chuang@ucsf.edu).

Running Title: mTORC1 signaling, mitochondrial capacity and lung development

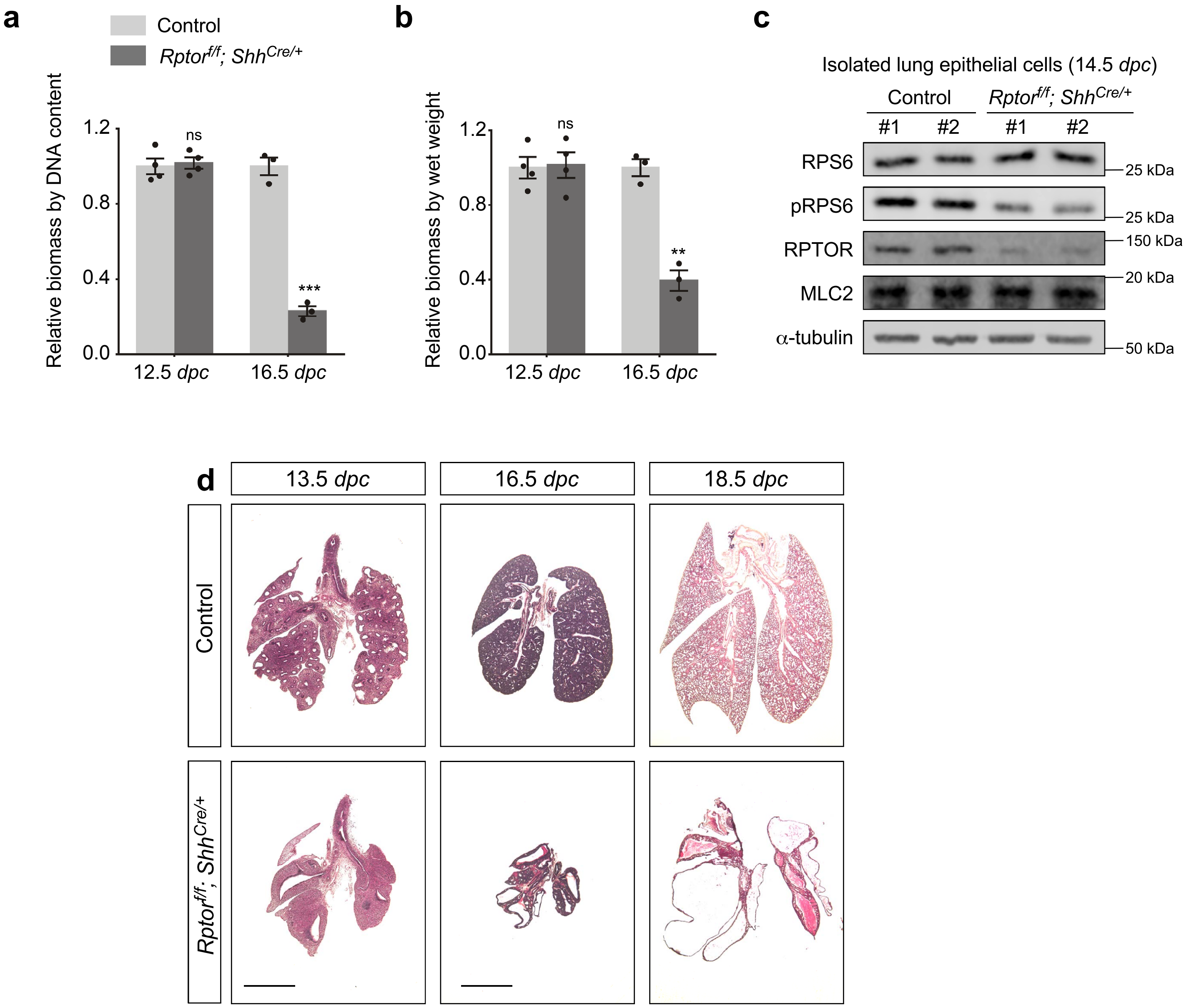

Supplementary Fig. 1. Characterization of *Rptor*-deficient lungs

(**a**, **b**) Measurement of lung biomass in control and *Rptor*$^{f/f}$; *Shh*$^{Cre/+}$ mice by either DNA content or wet weight at 12.5 (n = 4 for each group) or 16.5 (n = 3 for each group) *days post coitus* (*dpc*). (**c**) Western blotting of purified lung epithelial cells from control and *Rptor*$^{f/f}$; *Shh*$^{Cre/+}$ mice at 14.5 *dpc*. A reduction in RPTOR and pRPS6 levels reflects efficient removal of *Rptor* from the lung epithelium by *Shh*^{Cre}. The numbers indicate the molecular weight of proteins in kDa. (**d**) Hematoxylin and eosin (H&E) staining of lung sections from control and *Rptor*$^{f/f}$; *Shh*$^{Cre/+}$ mice at the stages indicated. Scale bars: 13.5 *dpc*, 0.5 mm; 16.5 and 18.5 *dpc*, 1 mm. All values are mean ± SEM. (**) p<0.01; (***) p<0.001; ns, not significant (two-tailed, unpaired Student's *t*-test). Source data are provided as a Source Data file.

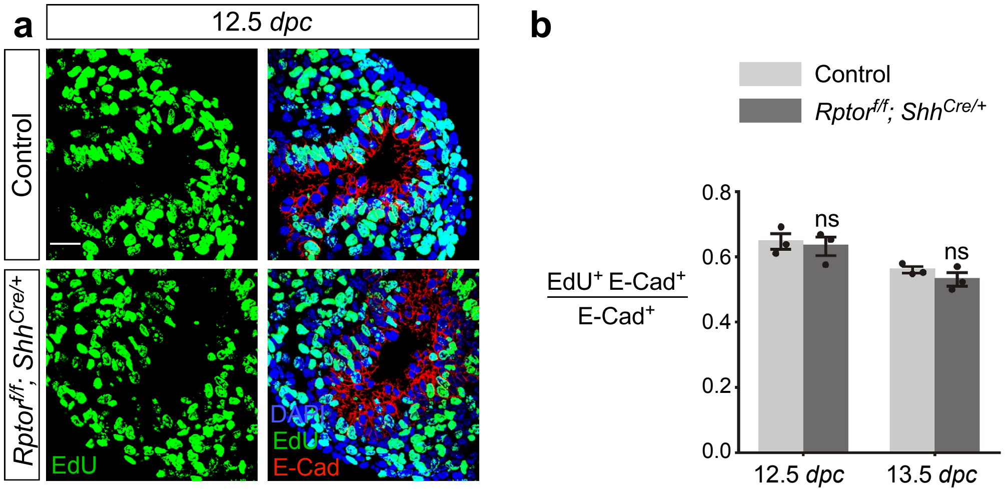

Supplementary Fig. 2. Measurement of cell proliferation in the absence of *Rptor*
(**a**) Immunostaining of lung sections collected from *Rptor*$^{f/f}$*; Shh*$^{Cre/+}$ mice and control littermates injected with EdU at 12.5 *days post coitus* (*dpc*). Lung epithelial cells were distinguished by E-Cad staining. Scale bar = 25 μm. (**b**) Quantification of epithelial cell proliferation in *Rptor*$^{f/f}$*; Shh*$^{Cre/+}$ mice and control littermates at 12.5 (n = 3 for each group) and 13.5 (n = 3 for each group) *dpc*. The rate of epithelial cell proliferation was calculated as the ratio of the number of EdU^{+} E-Cad^{+} cells to the number of E-Cad^{+} cells. No apparent alteration in the percentage of proliferating cells was detected in the absence of *Rptor*. All values are mean ± SEM. ns, not significant (two-tailed, unpaired Student's *t*-test). Source data are provided as a Source Data file.

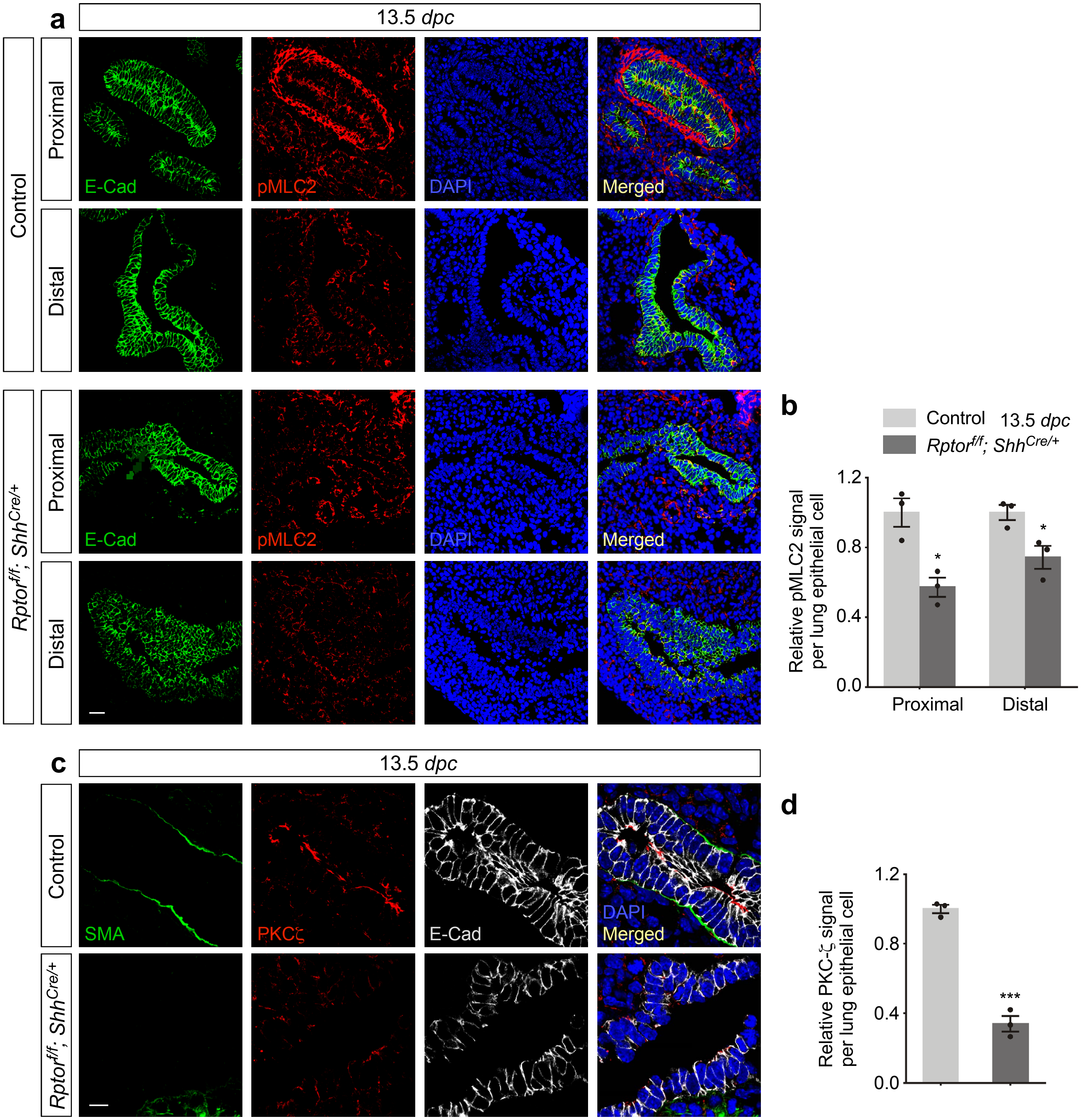

Supplementary Fig. 3. Loss of *Rptor* in the lung epithelium leads to reduced expression of pMLC2 and PKCζ

(**a**) Immunostaining of lung sections collected from control and *Rptor*$^{f/f}$*; Shh*$^{Cre/+}$ mice at 13.5 *days post coitus* (*dpc*). Both the proximal and distal airways were shown. E-Cadherin (E-Cad) labeled epithelial cells. Expression of phosphorylated myosin light chain 2 (pMLC2) is indicative of mechanical force production.Scale bar = 25 μm. (**b**) Quantification of pMLC2 signal in lung epithelial cells (n = 3 for each group). (**c**) Immunostaining of lung sections collected from control and *Rptor*$^{f/f}$*; Shh*$^{Cre/+}$ mice at 13.5 *dpc*. SMA, smooth muscle actin. Protein kinase C zeta (PKCζ) marked the apical surface of epithelial cells. Scale bar = 10 μm. (**d**) Quantification of PKCζ signal in lung epithelial cells (n = 3 for each group). All values are mean ± SEM. (*) p<0.05; (***) p<0.001 (two-tailed, unpaired Student's *t*-test). Source data are provided as a Source Data file.

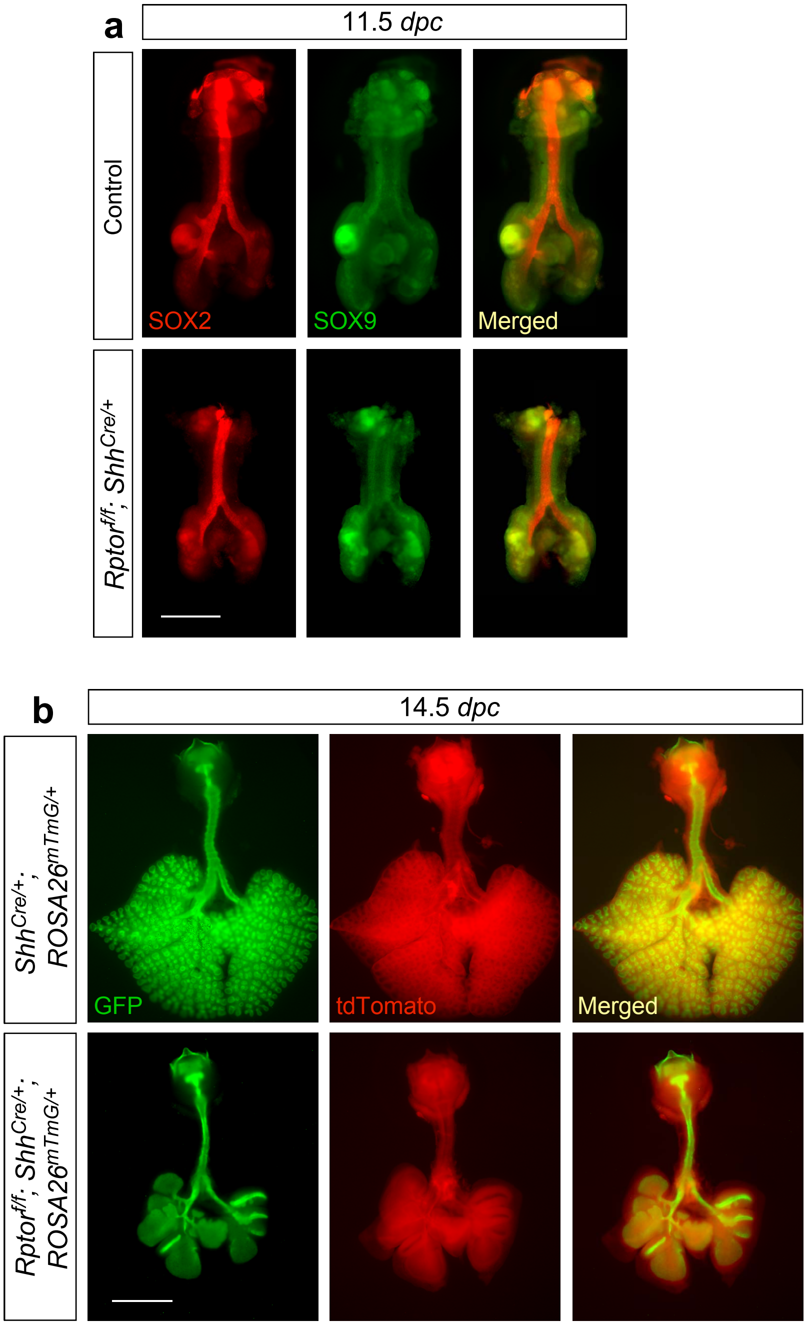

Supplementary Fig. 4. Disruption of SOX9–SOX2 distribution in *Rptor*-deficient lungs is apparent at early stages of lung branching

(**a**) Whole-mount immunostaining of dissected lungs from control and *Rptor*$^{f/f}$*; Shh*$^{Cre/+}$ mice at 11.5 *days post coitus* (*dpc*). SOX2 expression was confined to the trachea and main stem bronchi in the mutant lungs. Scale bar = 0.5 mm. (**b**) Whole-lung imaging of dissected lungs from *Shh*$^{Cre/+}$*; ROSA26*$^{mTmG/+}$ (control) and *Rptor*$^{f/f}$*; Shh*$^{Cre/+}$*; ROSA26*$^{mTmG/+}$ mice at 14.5 *dpc*. Scale bar = 1 mm.

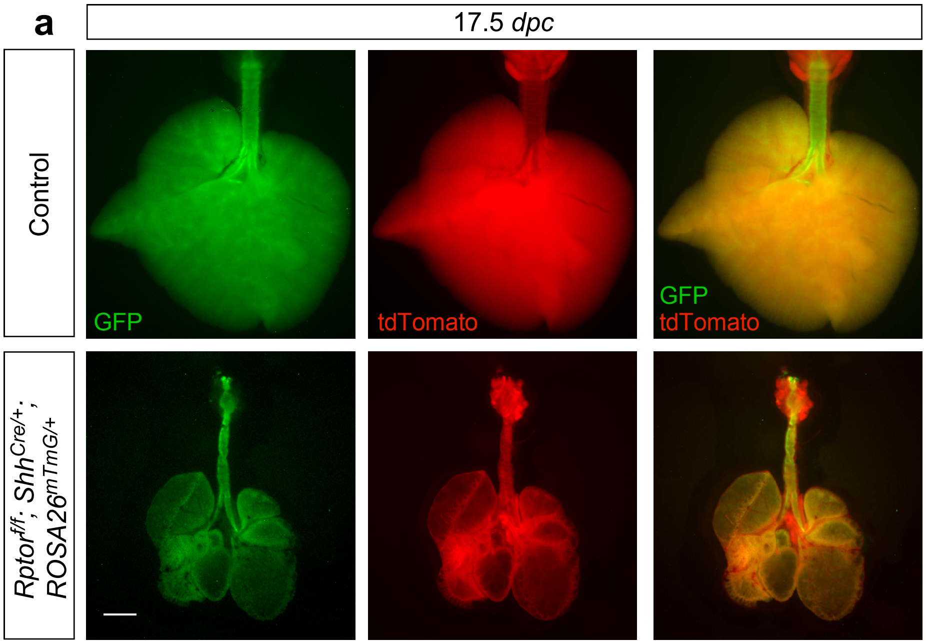

Supplementary Fig. 5. *Rptor* loss does not disrupt sacculation
(**a**) Whole-lung imaging of dissected lungs from $Shh^{Cre/+}$; $ROSA26^{mTmG/+}$ (control) and $Rptor^{f/f}$; $Shh^{Cre/+}$; $ROSA26^{mTmG/+}$ mice at 17.5 *days post coitus* (*dpc*). GFP was activated from the *ROSA26* allele in all lung epithelial cells by Shh^{Cre}; tdTomato marked all non-epithelial cells. Many GFP^{+} dots were present on the surface of $Rptor^{f/f}$; $Shh^{Cre/+}$; $ROSA26^{mTmG/+}$ lungs. Scale bar = 1 mm.

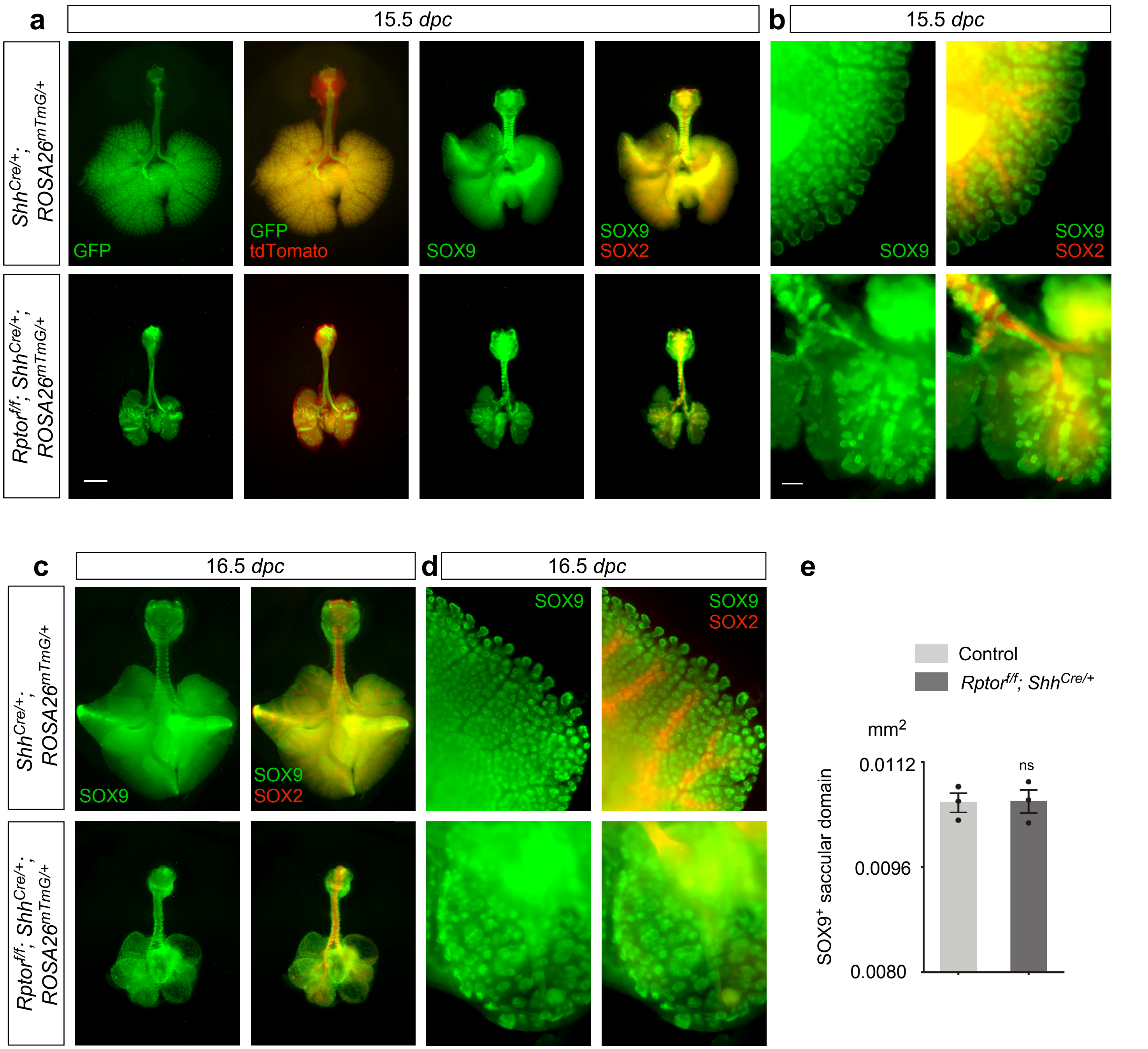

Supplementary Fig. 6. Saccule development still proceeds in *Rptor*-deficient lungs defective in forming the conducting airways

(**a**, **b**) Whole-mount immunostaining or whole-lung imaging of dissected lungs from *Shh*$^{Cre/+}$; *ROSA26*$^{mTmG/+}$ (control) and *Rptor*$^{f/f}$; *Shh*$^{Cre/+}$; *ROSA26*$^{mTmG/+}$ mice at 15.5 *days post coitus* (*dpc*). Scale bars: **a**, 1 mm; **b**, 100 μm. (**c**, **d**) Whole-mount immunostaining of dissected lungs from *Shh*$^{Cre/+}$; *ROSA26*$^{mTmG/+}$ (control) and *Rptor*$^{f/f}$; *Shh*$^{Cre/+}$; *ROSA26*$^{mTmG/+}$ mice at 16.5 *dpc*. Scale bars: **c**, 1 mm; **d**, 100 μm. (**e**) Measurement of SOX9^{+} saccular domains in control and *Rptor*-deficient lungs (n = 3 for each group). All values are mean ± SEM. ns, not significant (two-tailed, unpaired Student's *t*-test). Source data are provided as a Source Data file.

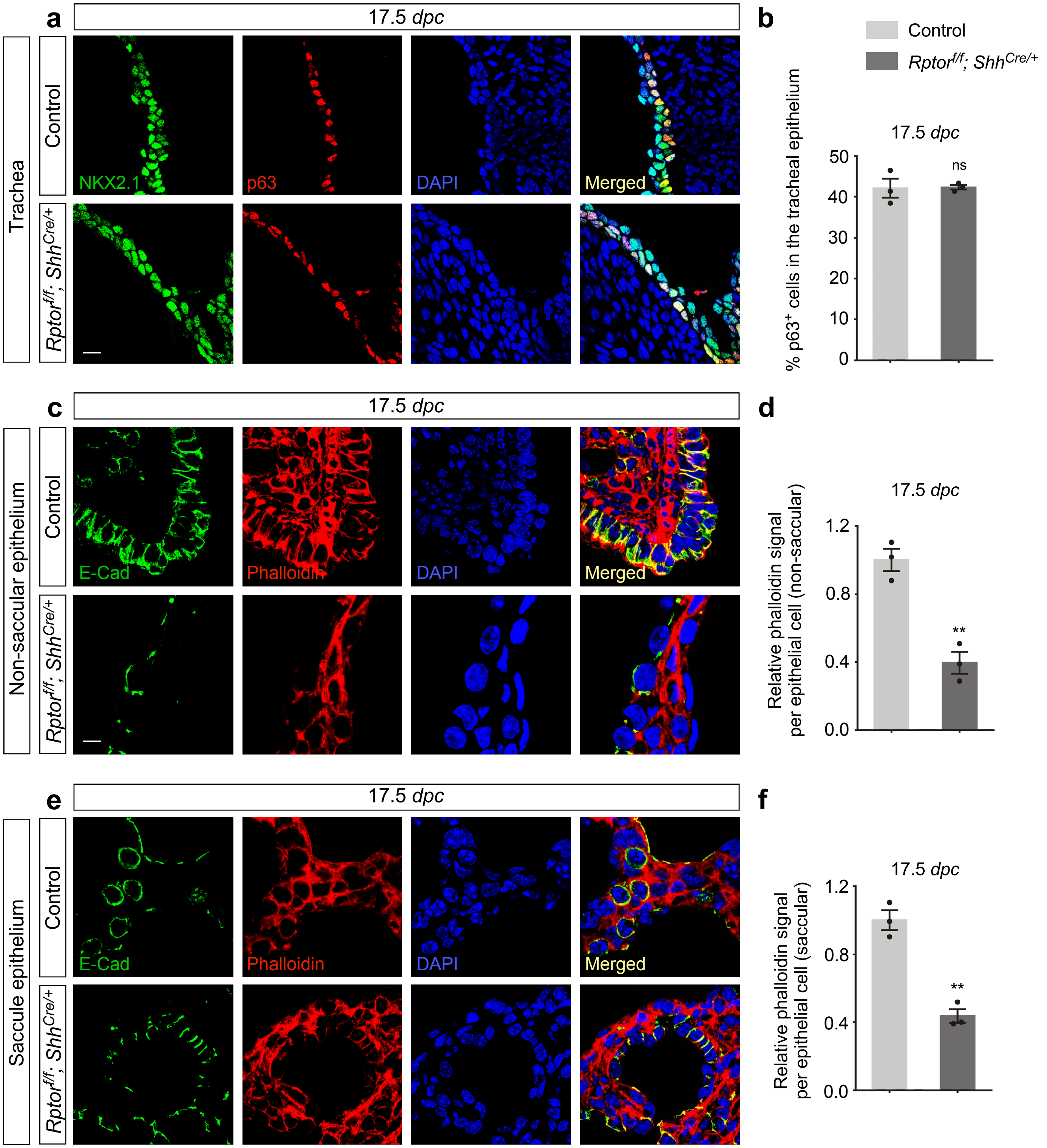

Supplementary Fig. 7. Loss of epithelial *Rptor* in the lungs disrupts proper proximal-distal patterning

(**a**) Immunostaining of lung sections collected from control and *Rptor*$^{f/f}$*; Shh*$^{Cre/+}$ mice at 17.5 *days post coitus* (*dpc*). NKX2.1 labeled all lung epithelial cell lineages; p63 is a marker for basal cells. Scale bar = 10 μm. (**b**) Quantification of p63^{+} cells in the trachea (n = 3 for each group). (**c**, **e**) Immunostaining of lung sections collected from control and *Rptor*$^{f/f}$*; Shh*$^{Cre/+}$ mice at 17.5 *dpc*. E-Cadherin (E-Cad) marked epithelial cells; phalloidin binds F-actin. Scale bar = 10 μm. (**d**) Quantification of phalloidin signal in the non-saccular epithelium (n = 3 for each group). (**f**) Quantification of phalloidin signal in the saccular epithelium (n = 3 for each group). All values are mean ± SEM. (**) p<0.01; ns, not significant (two-tailed, unpaired Student's *t*-test). Source data are provided as a Source Data file.

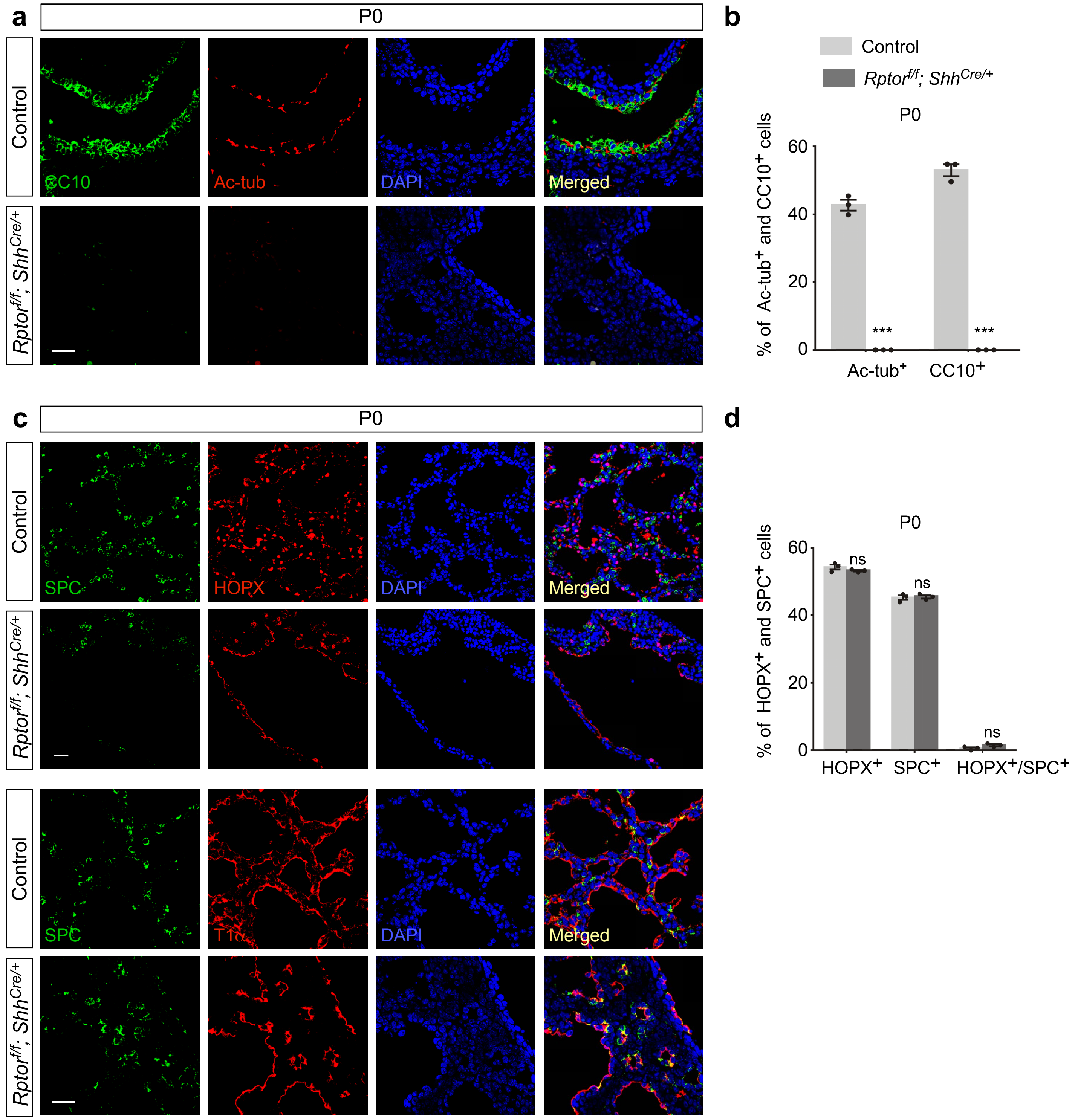

Supplementary Fig. 8. The conducting airways fail to form but saccule development proceeds in the absence of epithelial *Rptor*

(**a**) Immunostaining of lung sections collected from control and *Rptor$^{f/f}$; Shh$^{Cre/+}$* mice at postnatal (P) day 0. CC10 labeled club cells; Ac-tub marked ciliated cells. Scale bar = 25 μm. (**b**) Quantification of CC10^{+} and Ac-tub^{+} cells (n = 3 for each group). (**c**) Immunostaining of lung sections collected from control and *Rptor$^{f/f}$; Shh$^{Cre/+}$* mice at P0. SPC is a marker for alveolar type II (AT2) cells; HOPX and T1α are markers for alveolar type I (AT1) cells. Lung saccules contain AT1 and AT2 cells. Scale bar = 25 μm for SPC/HOPX staining; scale bar = 25 μm for SPC/T1α staining. (**d**) Quantification of SPC^{+} and HOPX^{+} cells (n = 3 for each group). All values are mean ± SEM. (***) p<0.001; ns, not significant (two-tailed, unpaired Student's *t*-test). Source data are provided as a Source Data file.

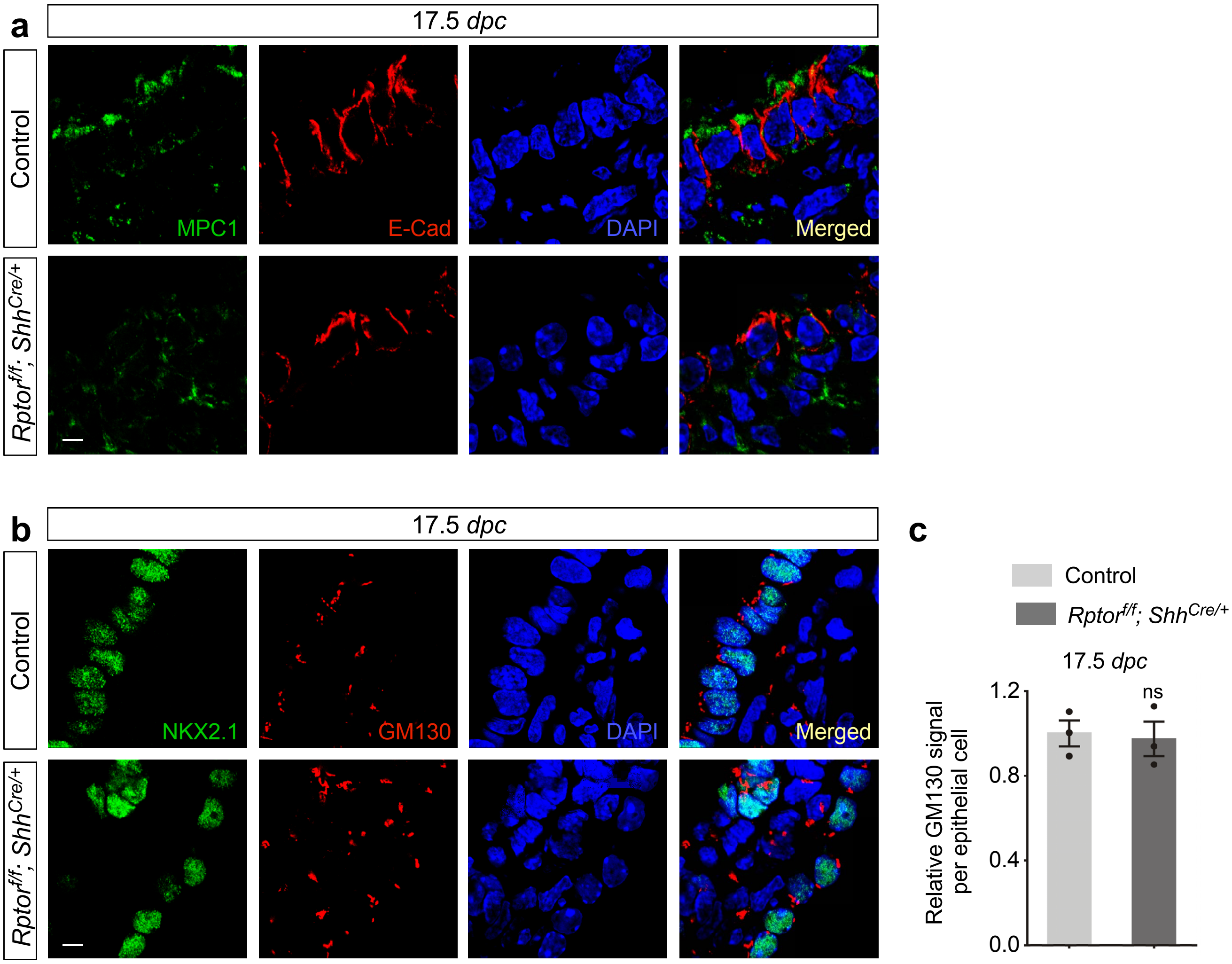

Supplementary Fig. 9. Perturbed mTORC1 signaling in the lung epithelium results in a reduction in mitochondrial number but does not affect the Golgi apparatus

(**a**, **b**) Immunostaining of lung sections collected from control and *Rptor*$^{f/f}$*; Shh*$^{Cre/+}$ mice at 17.5 *days post coitus* (*dpc*). NKX2.1 labeled all lung epithelial cell lineages; E-Cadherin (E-Cad) marked epithelial cells; MPC1 is a marker for mitochondria; GM130 is a marker for the Golgi apparatus. Scale bar = 5 μm. (**c**) Quantification of GM130 signal in the lung epithelium (n = 3 for each group). All values are mean ± SEM. ns, not significant (two-tailed, unpaired Student's *t*-test). Source data are provided as a Source Data file.

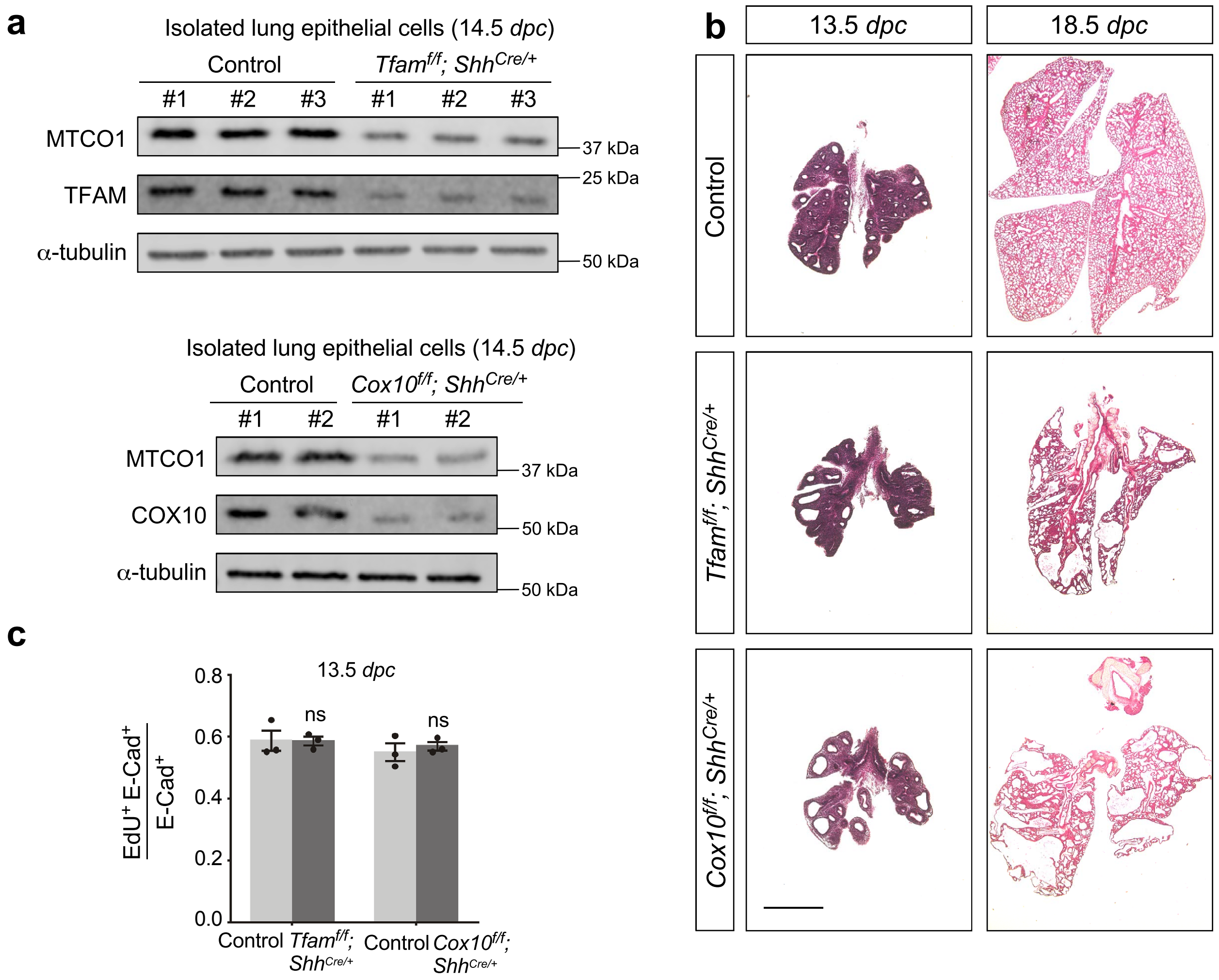

Supplementary Fig. 10. Characterization of *Tfam*- and *Cox10*-deficient lungs

(**a**) Western blotting of purified lung epithelial cells from control and *Tfam$^{f/f}$; Shh$^{Cre/+}$ and Cox10$^{f/f}$; Shh$^{Cre/+}$* mice at 14.5 *dpc*. A reduction in TFAM and COX10 levels reflects efficient removal of *Tfam or Cox10* from the lung epithelium by *Shh^{Cre}*. The numbers indicate the molecular weight of proteins in kDa. (**b**) Hematoxylin and eosin (H&E) staining of lung sections from control, *Tfam$^{f/f}$; Shh$^{Cre/+}$* and *Cox10$^{f/f}$; Shh$^{Cre/+}$* mice at 13.5 and 18.5 *days post coitus* (*dpc*). Scale bar = 1 mm. (**c**) Quantification of epithelial cell proliferation in lungs from control, *Tfam$^{f/f}$; Shh$^{Cre/+}$* and *Cox10$^{f/f}$; Shh$^{Cre/+}$* mice at 13.5 *dpc* (n = 3 for each group). The rate of epithelial cell proliferation was calculated as the ratio of the number of EdU$^+$ E-Cad$^+$ cells to the number of E-Cad$^+$ cells. No apparent alteration in the percentage of proliferating cells was detected in the absence of *Tfam* or *Cox10*. All values are mean ± SEM. ns, not significant (two-tailed, unpaired Student's *t*-test). Source data are provided as a Source Data file.

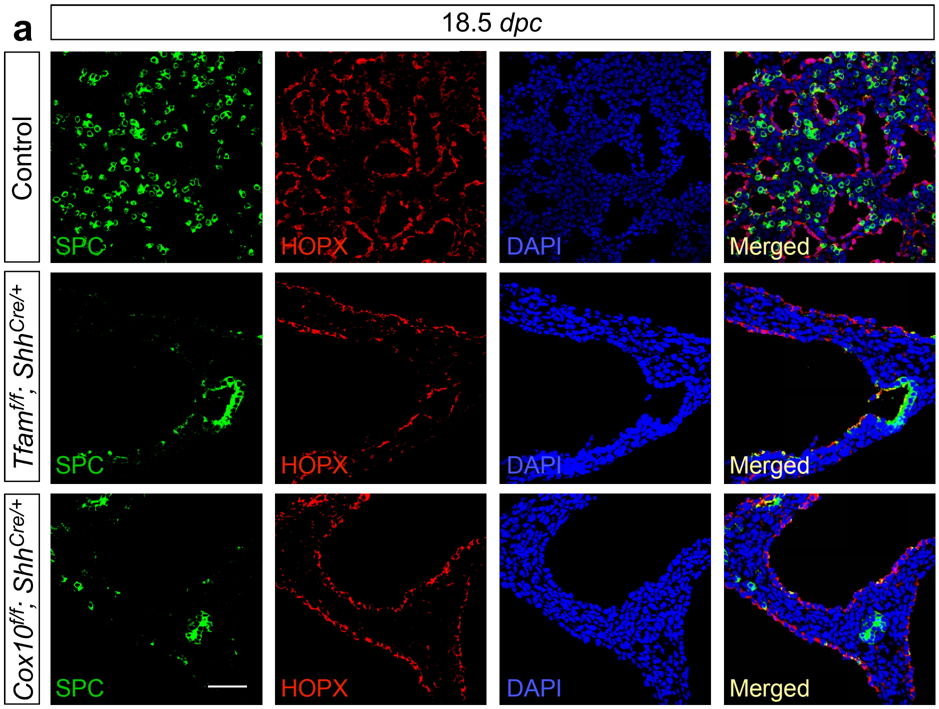

Supplementary Fig. 11. Characterization of *Tfam*- and *Cox10*-deficient lungs
(**a**) Immunostaining of lung sections collected from control, *Tfam*$^{f/f}$; *Shh*$^{Cre/+}$ and *Cox10*$^{f/f}$; *Shh*$^{Cre/+}$ mice at 18.5 *days post coitus* (*dpc*). SPC labeled alveolar type II cells; HOPX marked alveolar type I cells. Scale bar = 50 μm.

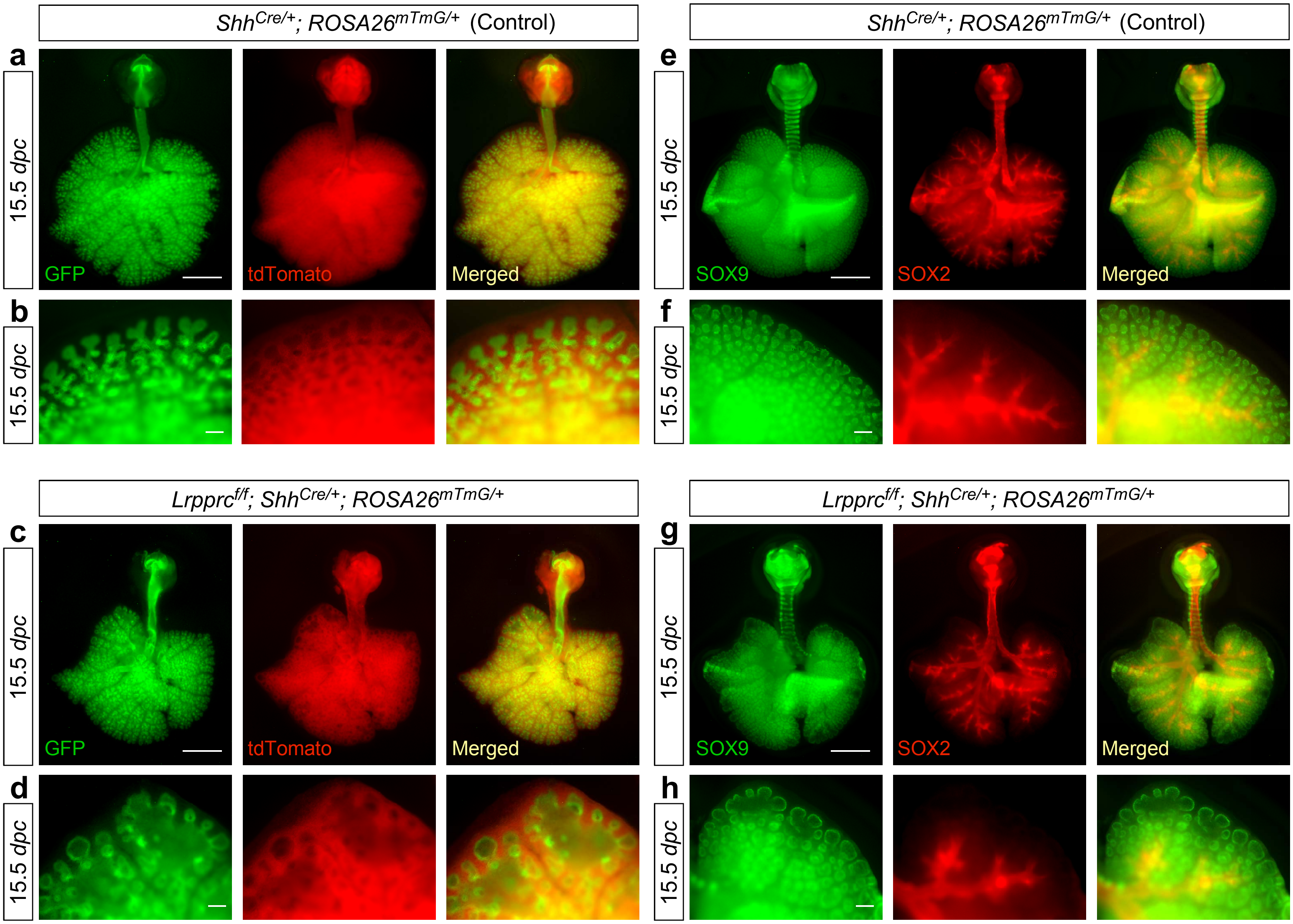

Supplementary Fig. 12. Ablation of epithelial *Lrpprc* disrupts the formation of the conducting airways (**a**-**d**) Whole-lung imaging of dissected lungs from *Shh*$^{Cre/+}$*; ROSA26*$^{mTmG/+}$ (control) and *Lrpprc*$^{f/f}$*; Shh*$^{Cre/+}$*; ROSA26*$^{mTmG/+}$ mice at 15.5 *days post coitus* (*dpc*). Scale bars: **a**, **c**, 1 mm; **b**, 100 μm; **d**, 143 μm. (**e**-**h**) Whole-mount immunostaining of dissected lungs from *Shh*$^{Cre/+}$*; ROSA26*$^{mTmG/+}$ (control) and *Lrpprc*$^{f/f}$*; Shh*$^{Cre/+}$*; ROSA26*$^{mTmG/+}$ mice at 15.5 *dpc*. Scale bars: **e**, **g**, 1 mm; **f**, 100 μm; **h**, 143 μm.

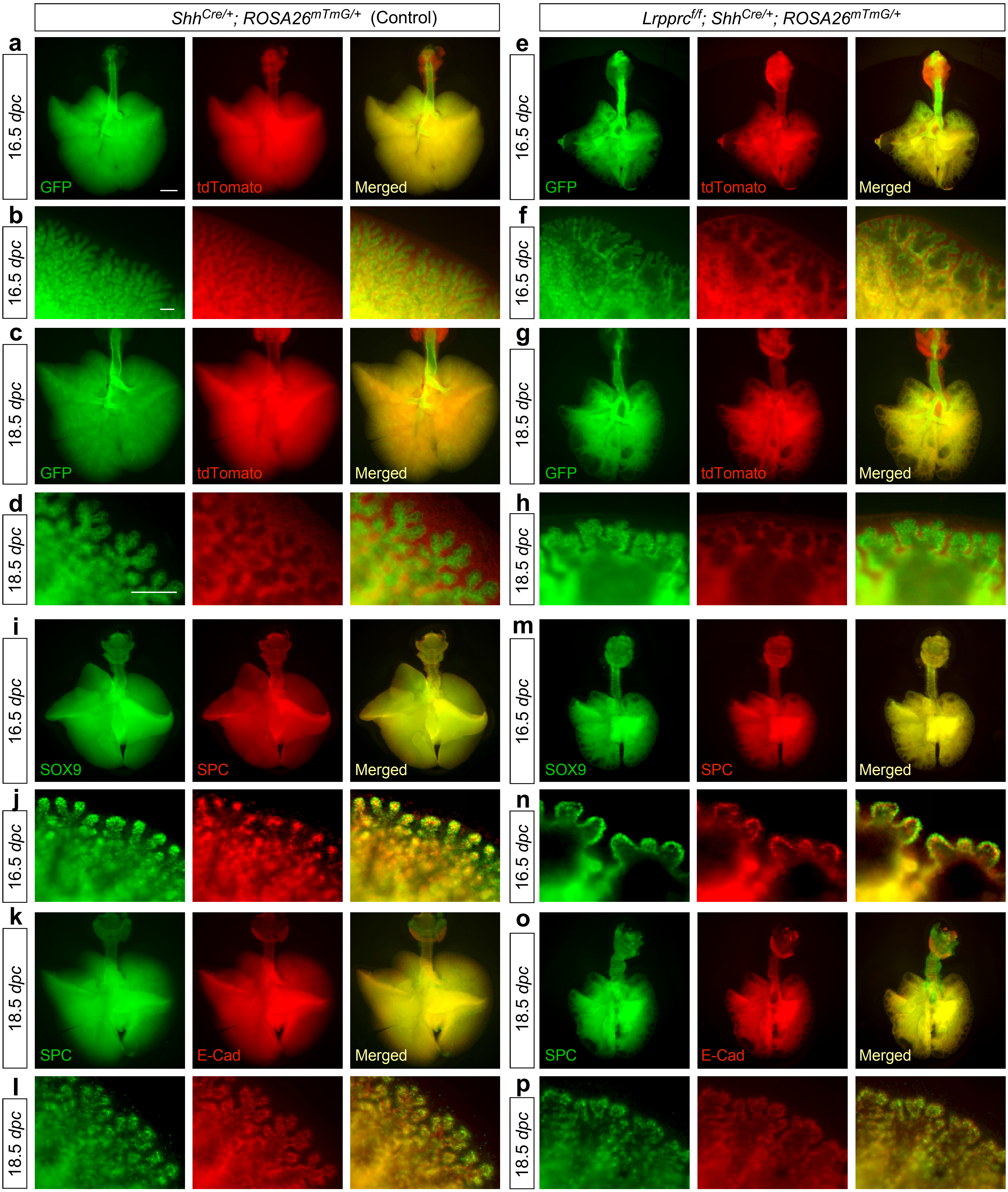

Supplementary Fig. 13. Saccule-like structures still form in the absence of epithelial *Lrpprc*
(**a**-**p**) Whole-lung imaging or whole-mount immunostaining of dissected lungs from *Shh*$^{Cre/+}$*; ROSA26*$^{mTmG/+}$ (control) and *Lrpprc*$^{f/f}$*; Shh*$^{Cre/+}$*; ROSA26*$^{mTmG/+}$ mice at 16.5 and 18.5 *days post coitus* (*dpc*). E-Cad marked epithelial cells; SOX9 labeled the distal lung progenitors; SPC labeled alveolar type II cells. Scale bars: **a**, **c**, **e**, **g**, **i**, **k**, **m**, **o**, 1 mm; **b**, **f**, 100 μm; **d**, **h**, **j**, **l**, **n**, **p**, 0.5 mm.

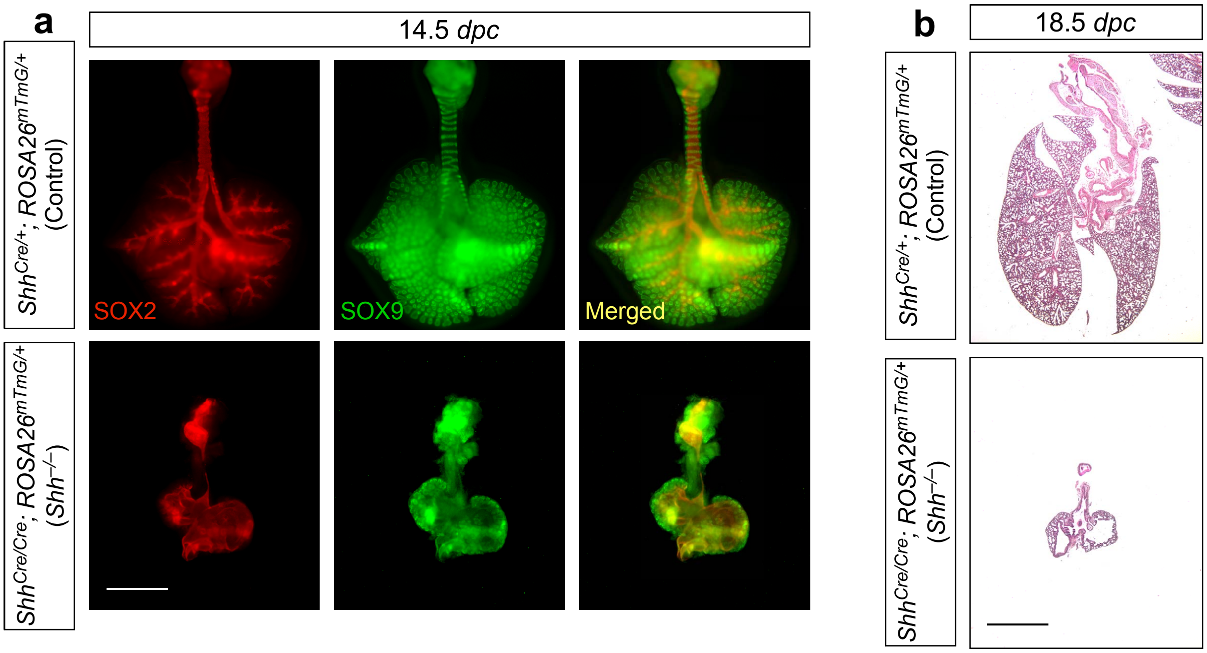

Supplementary Fig. 14. Characterization of *Shh*-deficient lungs

(**a**) Whole-mount immunostaining of dissected lungs from $Shh^{Cre/+}$; $ROSA26^{mTmG/+}$ (control) and $Shh^{Cre/Cre}$; $ROSA26^{mTmG/+}$ ($Shh^{-/-}$) lungs at 14.5 *days post coitus* (*dpc*). SOX2 labeled proximal airway epithelium; SOX9 marked distal airway epithelium. Scale bar = 1 mm. (**b**) Hematoxylin and eosin (H&E) staining of lung sections from control and $Shh^{-/-}$ lungs at 18.5 *dpc*. Scale bar = 1 mm.

a

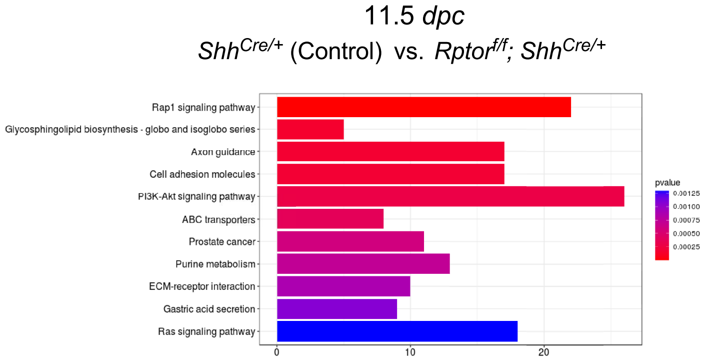

b

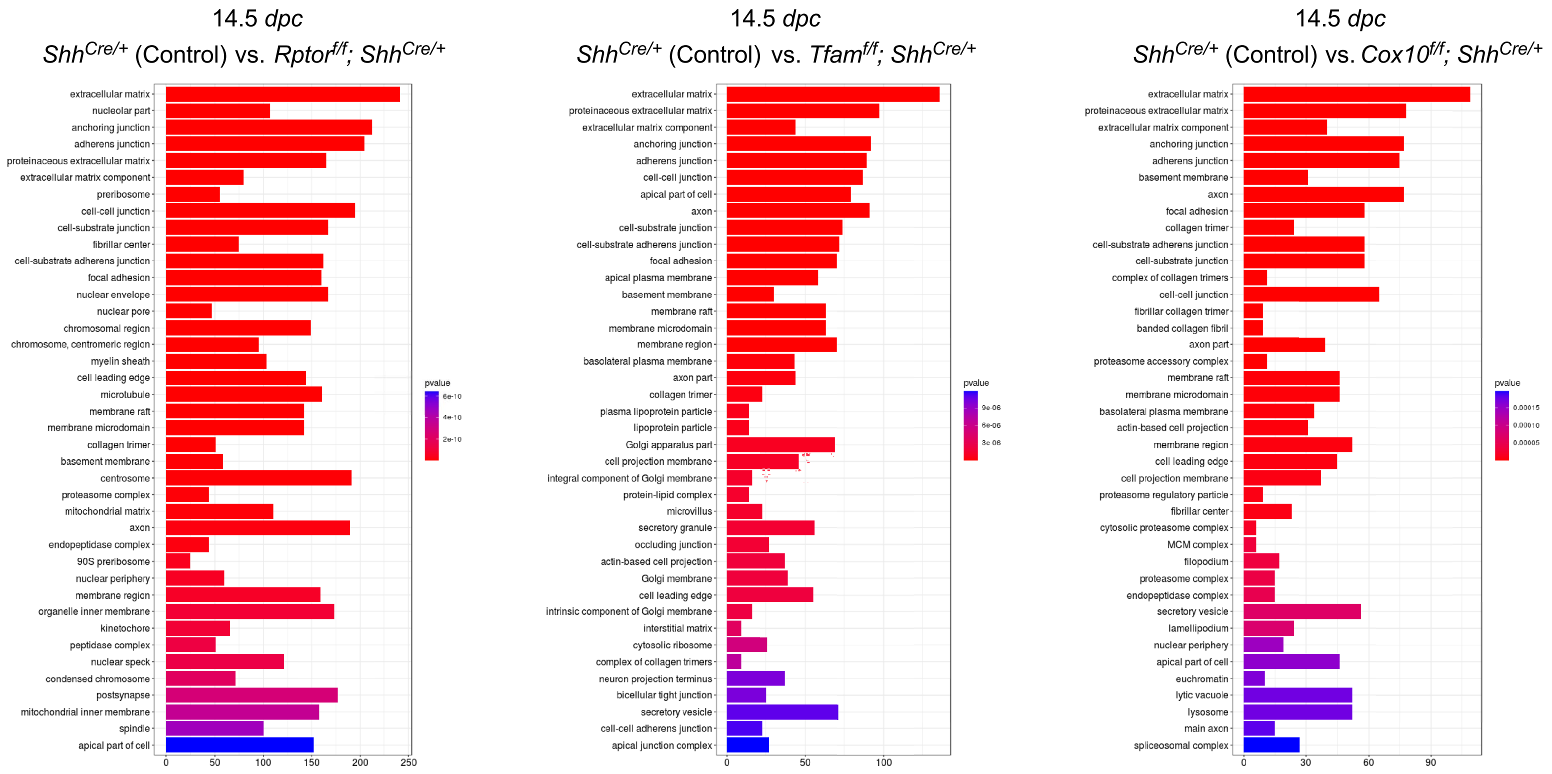

Supplementary Fig. 15. RNA-seq analysis of *Rptor*, *Tfam* and *Cox10* mutant lungs
(**a**) KEGG pathway analysis of bulk RNA-seq analysis of control and *Rptor*$^{f/f}$*; Shh*$^{Cre/+}$ lungs at 11.5 *days post coitus* (*dpc*). (**b**) Gene Ontology (GO) pathway analysis of bulk RNA-seq analysis of control, *Rptor*$^{f/f}$*; Shh*$^{Cre/+}$, *Tfam*$^{f/f}$*; Shh*$^{Cre/+}$ and *Cox10*$^{f/f}$*; Shh*$^{Cre/+}$ lungs at 14.5 *dpc*. The top 40 enriched terms in GO Cellular Component were shown. The mitochondrial pathway was not among the top 40 enriched pathways. This is likely because *Rptor* controls mitochondrial genes posttranscriptionally.
