# Supplementary material for: mTORC1 signaling facilitates differential stem cell differentiation to shape the developing murine lung and is associated with mitochondrial capacity
Source: Nat Commun. 2022 Nov 25;13:7252. doi: 10.1038/s41467-022-34763-y (PMC9700781; doi:10.1038/s41467-022-34763-y)
Supplement: Supplementary file 3 — Reporting Summary [file 41467_2022_34763_MOESM3_ESM.pdf]

nature portfolio

Corresponding author(s): Pao-Tien Chuang

Last updated by author(s): Nov 2, 2022

Reporting Summary

Nature Portfolio wishes to improve the reproducibility of the work that we publish. This form provides structure for consistency and transparency in reporting. For further information on Nature Portfolio policies, see our Editorial Policies and the Editorial Policy Checklist.

Statistics

For all statistical analyses, confirm that the following items are present in the figure legend, table legend, main text, or Methods section.

n/a	Confirmed	
☐	☒	The exact sample size (*n*) for each experimental group/condition, given as a discrete number and unit of measurement
☐	☒	A statement on whether measurements were taken from distinct samples or whether the same sample was measured repeatedly
☐	☒	The statistical test(s) used AND whether they are one- or two-sided<br>*Only common tests should be described solely by name; describe more complex techniques in the Methods section.*
☒	☐	A description of all covariates tested
☒	☐	A description of any assumptions or corrections, such as tests of normality and adjustment for multiple comparisons
☐	☒	A full description of the statistical parameters including central tendency (e.g. means) or other basic estimates (e.g. regression coefficient) AND variation (e.g. standard deviation) or associated estimates of uncertainty (e.g. confidence intervals)
☐	☒	For null hypothesis testing, the test statistic (e.g. *F*, *t*, *r*) with confidence intervals, effect sizes, degrees of freedom and *P* value noted<br>*Give P values as exact values whenever suitable.*
☒	☐	For Bayesian analysis, information on the choice of priors and Markov chain Monte Carlo settings
☒	☐	For hierarchical and complex designs, identification of the appropriate level for tests and full reporting of outcomes
☒	☐	Estimates of effect sizes (e.g. Cohen's *d*, Pearson's *r*), indicating how they were calculated

Our web collection on statistics for biologists contains articles on many of the points above.

Software and code

Policy information about availability of computer code

Data collection	Confocal images were captured on a Leica SPE laser-scanning confocal microscope;<br>Whole-mount immunostaining and whole-lung imaging were captured on a Nikon Eclipse E1000 microscope with a SPOT 2.3 CCD camera;<br>Quantitative PCR was performed on an Applied Biosystems QuantStudioTM 5 Real-Time PCR System;<br>Library quality of bulk RNA-Seq was evaluated with an Agilent 2100 Bioanalyzer;<br>Library of bulk RNA-Seq was sequenced on BGISEQ-500 platform.<br>Western blot images were captured on a LI-COR Odyssey Fc device.<br>Measurement of the diameter, distance, thickness, area and the percentage of the cell were performed using ImageJ (Version 1.49).
Data analysis	Confocal images adjustments (red/green/blue/grey histograms and channel merges) were performed using LAS AF Lite;<br>Adjustment of the color and lightness of the whole-mount immunostaining and whole-lung imaging pictures was performed using ImageJ;<br>EdU incorporation related quantification were performed using ImageJ;<br>The graph was generated using GraphPad Prism 7;<br>Differential gene expression, gene ontology (GO) enrichment analyses and the barplot of gene ontology enrichment were performed with RStudio, R version 3.4.0;<br>Heatmap images were generated using online Heatmapper software.

For manuscripts utilizing custom algorithms or software that are central to the research but not yet described in published literature, software must be made available to editors and reviewers. We strongly encourage code deposition in a community repository (e.g. GitHub). See the Nature Portfolio guidelines for submitting code & software for further information.

Data

Policy information about availability of data

All manuscripts must include a data availability statement. This statement should provide the following information, where applicable:

- Accession codes, unique identifiers, or web links for publicly available datasets
- A description of any restrictions on data availability
- For clinical datasets or third party data, please ensure that the statement adheres to our policy

All the related data for this study are available in the published article and the supplementary information file. Additional data that support the findings of this study are available from the corresponding authors upon reasonable request. These additional data are mainly images that are identical to the published images but were taken at a different color channel. They do not provide essential new information to this manuscript.
Raw and analyzed data of RNA-Seq have been deposited to the Gene Expression Omnibus (GEO) database under accession numbers GSE189327 [https://www.ncbi.nlm.nih.gov/geo/query/acc.cgi?acc=GSE189327] and GSE213202 [https://www.ncbi.nlm.nih.gov/geo/query/acc.cgi?acc=GSE213202].
Source data are provided with this paper.

Field-specific reporting

Please select the one below that is the best fit for your research. If you are not sure, read the appropriate sections before making your selection.

☒ Life sciences ☐ Behavioural & social sciences ☐ Ecological, evolutionary & environmental sciences

For a reference copy of the document with all sections, see nature.com/documents/nr-reporting-summary-flat.pdf

Life sciences study design

All studies must disclose on these points even when the disclosure is negative.

Sample size	For all the in vivo experiments, at least three biological repeats were performed. The sample size was indicated in the main text and figure legends. We did not use statistical methods to predetermine the sample size.The mutant phenotypes described in this study were completely penetrant. All the mice of the same genotype showed similar, if not identical, lung phenotypes at each time point and there was little variation between the mutant lungs. The n numbers used in this study provide adequate statistical significance.
Data exclusions	No data were excluded in this study.
Replication	The replication numbers were included in the corresponding figure legends.
Randomization	The control and mutant mouse lungs that we reported in this study were based on the genotyping results, and both sexes were included in all time points.
Blinding	Blinding was irrelevant for mouse work in this study since the experimental groups were assigned based on the genotyping results.<br>For bulk RNA-Seq library preparation and sequencing, the investigators were blinded to the processing conditions.

Reporting for specific materials, systems and methods

We require information from authors about some types of materials, experimental systems and methods used in many studies. Here, indicate whether each material, system or method listed is relevant to your study. If you are not sure if a list item applies to your research, read the appropriate section before selecting a response.

Materials & experimental systems

n/a	Involved in the study	
☐	☒	Antibodies
☒	☐	Eukaryotic cell lines
☒	☐	Palaeontology and archaeology
☐	☒	Animals and other organisms
☒	☐	Human research participants
☒	☐	Clinical data
☒	☐	Dual use research of concern

Methods

n/a	Involved in the study	
☒	☐	ChIP-seq
☒	☐	Flow cytometry
☒	☐	MRI-based neuroimaging

Antibodies

Antibodies used	chicken anti-GFP (1:200, abcam, Cat# ab13970; RRID:AB_300798)<br>rabbit anti-NKX2.1 (1:100, Epitomics, Cat# 2044–1; RRID:AB_1267367)<br>goat anti-CC10 (1:200, Santa Cruz Biotechnology, Cat# sc-9773; RRID:AB_2183391)

mouse anti-acetylated tubulin (1:200, MilliporeSigma, Cat# T6793; RRID:AB_477585)
rabbit anti-prosurfactant protein C (proSP-C) (1:200, MilliporeSigma, Cat# AB3786; RRID:AB_91588)
hamster anti-T1α (1:200, Developmental Studies Hybridoma Bank, Cat# 8.1.1; RRID:AB_531893)
mouse anti-HOPX (1:100, Santa Cruz Biotechnology, Cat# sc-398703; RRID:AB_2687966)
mouse anti-p63 (1:100, Santa Cruz Biotechnology, Cat# sc-8431; RRID:AB_628091)
rabbit anti-MPC1 (1:100, MilliporeSigma, Cat# HPA045119; RRID:AB_10960421)
rat anti-E cadherin (1:200, Life Technologies, Cat# 13-1900; RRID:AB_2533005)
mouse anti-β-catenin (1:100, BD Transduction Laboratories, Cat# 610154; RRID:AB_397555)
mouse anti-MTCO1 (1:100, abcam, Cat# ab14705; RRID:AB_2084810)
mouse anti-GM130 (1:100, BD Biosciences, Cat# 610822; RRID:AB_398141)
rabbit anti-MLC2 (1:500; Cell Signaling Technology, Cat# 3672; RRID:AB_10692513)
goat anti-TFAM (1: 250, Santa Cruz Biotechnology, Cat# sc-23588; RRID:AB_2303230)
rabbit anti-COX10 (1:500, Proteintech, Cat# 10611-2-AP; RRID:AB_2084833)
mouse anti-alpha-tubulin (1:3000, Developmental Studies Hybridoma Bank, Cat# 12G10; RRID:AB_1157911)
mouse anti-ACTA2 (1:200, Thermo Scientific Lab Vision, Cat# MS-113-P0; RRID:AB_64001)
rabbit anti-Raptor (1:2000, EMD Millipore Corporation, Cat# 09-217; RRID:AB_612103)
rat anti-PECAM-1 (CD31) (1:150, Santa Cruz Biotechnology, Cat# sc-18916; RRID:AB_627028)
rabbit anti-PDGFRA (1:150, Cell Signaling Technology, Cat# 3164; RRID:AB_2162351)
mouse anti-S6 Ribosomal Protein (1:100, Cell Signaling Technology, Cat# 2317; RRID:AB_2238583)
rabbit anti-Phospho-S6 Ribosomal Protein (Ser235/236) (1:100, Cell Signaling Technology, Cat# 4856; RRID:AB_2181037)
rabbit anti-SOX2 (D9B8N) (1:200, Cell Signaling Technology, Cat# 23064; RRID:AB_2714146)
goat anti-SOX9 (1:200, R&D Systems, Cat# AF3075; RRID:AB_2194160)
rabbit anti-pMLC2 (S19) (1:100, Cell Signaling Technology, Cat# 3671; RRID:AB_330248)
rabbit anti-PKC Zeta (C-20) (1:100, Santa Cruz Biotechnology, Cat# sc-216; RRID:AB_2300359)
rat anti-CD326 (EpCAM) (G8.8) (1:100, eBioscience, Cat# 13-5791-82, RRID:AB_1659713).

Alexa Fluor® 488 donkey anti-chicken antibody (1:1000, Jackson ImmunoResearch Laboratories, Cat# 703-546-155; RRID:AB_2340376),
Alexa Fluor® 488 donkey anti-goat (1:1000, Life Technologies, Cat# A11055; RRID:AB_2534102),
Alexa Fluor® 488 donkey anti-mouse (1:1000, Life Technologies, Cat# A21202; RRID:AB_141607),
Alexa Fluor® 594 donkey anti-mouse (1:1000, Life Technologies, Cat# A21203; RRID:AB_141633),
Alexa Fluor® 647 donkey anti-mouse (1:1000, Life Technologies, Cat# A31571; RRID:AB_162542),
Alexa Fluor® 488 donkey anti-rabbit (1:1000, Life Technologies, Cat# A21206; RRID:AB_2535792),
Alexa Fluor® 594 donkey anti-rabbit (1:1000, Life Technologies, Cat# A21207; RRID:AB_141637),
Alexa Fluor® 594 donkey anti-rat (1:1000, Life Technologies, Cat# A21209; RRID:AB_2535795),
Biotinylated goat-anti hamster (1:1000, Vector Laboratories, Cat# BA-9100; RRID:AB_2336137),
Biotin-SP-conjugated AffiniPure donkey anti-rabbit (1:1000, Jackson ImmunoResearch Laboratories, Cat# 711-065-152; RRID:AB_2340593),
Biotin-SP-conjugated AffiniPure donkey anti-rat (1:1000, Jackson ImmunoResearch Laboratories, Cat# 712-065-150; RRID:AB_2340646),
Biotinylated horse anti-mouse (1:1000, Vector Laboratories, Cat# BA-2000; RRID:AB_2313581),
Streptavidin, Alexa Fluor® 488 conjugate antibody (1:1000, Life Technologies, Cat# S11223),
Streptavidin, Alexa Fluor® 594 conjugate antibody (1:1000, Life Technologies, Cat# S11227),
Streptavidin, Alexa Fluor® 647 conjugate antibody (1:1000, Jackson ImmunoResearch Laboratories, Cat# 016-600-084; RRID:AB_2341101)

Validation

All the antibodies in this study were purchased from commercial vendors. They have been widely used in mouse lungs and other tissues in publications from our lab and other groups.
e.g., Lin et al., 2017, eLife; Zhang et al., 2020, eLife; Zhang et al., 2022, Developmental Cell; Zhang et al., 2022, PLOS Biology.

Animals and other organisms

Policy information about studies involving animals; ARRIVE guidelines recommended for reporting animal research

Laboratory animals

The animals we used are:
mouse, Rptor floxed allele [B6.Cg-Rptortm1.1Dmsa/J]
mouse, Tfam floxed allele [B6.Cg-Tfamtm1.1Ncdl/J]
mouse, Cox10 floxed allele [B6.129X1-Cox10tm1Ctm/J]
mouse, Lrpprc floxed allele [Lrpprctm1.1Lrsn/J]
mouse, ROSA26mTmG allele [Gt(ROSA)26Sortm4(ACTB-tdTomato,-EGFP)Luo/J]
mouse, ROSA26tdTomato allele [B6;129S6-Gt(ROSA)26Sortm14(CAG-tdTomato)Hze/J]
mouse, ShhCre allele [B6.Cg-Shhtm1(EGFP/cre)Cjt/J]
Mice of both sexes at the breeding age were used for mating. Embryos of both sexes and at different developmental stages were collected for analysis.

Wild animals

No wild animals were used in this study.

Field-collected samples

No field-collected samples were used in this study.

Ethics oversight	The mouse experiments in this study were performed following the protocols (AN187712) approved by the Institutional Animal Care and Use Committee (IACUC) of the University of California, San Francisco (UCSF).

Note that full information on the approval of the study protocol must also be provided in the manuscript.
